# Exogenous α-synuclein hinders synaptic communication in cultured cortical primary rat neurons

**DOI:** 10.1371/journal.pone.0193763

**Published:** 2018-03-22

**Authors:** G. C. Hassink, C. C. Raiss, I. M. J. Segers-Nolten, R. J. A. van Wezel, V. Subramaniam, J. le Feber, M. M. A. E. Claessens

**Affiliations:** 1 Clinical Neurophysiology, MIRA Institute for Biomedical Technology and Technical Medicine, University of Twente, Postbus, Enschede, the Netherlands; 2 Biomedical Signal and Systems, MIRA Institute for Biomedical Technology and Technical Medicine, University of Twente, Postbus, Enschede, the Netherlands; 3 Nanobiophysics Group, MESA+ Institute for Nanotechnology, University of Twente, Postbus, Enschede, the Netherlands; 4 Biophysics, Donders Institute for Brain, Cognition and Behaviour, Radboud University, Nijmegen, Postbus, The Netherlands; Hertie Institute for Clinical Brain Research and German Center for Neurodegenerative Diseases, GERMANY

## Abstract

Amyloid aggregates of the protein α-synuclein (αS) called Lewy Bodies (LB) and Lewy Neurites (LN) are the pathological hallmark of Parkinson’s disease (PD) and other synucleinopathies. We have previously shown that high extracellular αS concentrations can be toxic to cells and that neurons take up αS. Here we aimed to get more insight into the toxicity mechanism associated with high extracellular αS concentrations (50–100 μM). High extracellular αS concentrations resulted in a reduction of the firing rate of the neuronal network by disrupting synaptic transmission, while the neuronal ability to fire action potentials was still intact. Furthermore, many cells developed αS deposits larger than 500 nm within five days, but otherwise appeared healthy. Synaptic dysfunction clearly occurred before the establishment of large intracellular deposits and neuronal death, suggesting that an excessive extracellular αS concentration caused synaptic failure and which later possibly contributed to neuronal death.

## Introduction

The three most common synucleinopathies are Parkinson's disease (PD), Lewy body dementia (LBD), and multiple system atrophy (MSA) [1]. In all these diseases, the protein α-synuclein (αS) aggregates into amyloid fibrils which are deposited in characteristic inclusions, i.e. Lewy bodies and Lewy neurites [1, 2]. The presence of Lewy bodies does, however, not always correlate with neurodegeneration and disease symptoms [3]. The relation between αS aggregation, the formation of αS amyloid inclusions, clearance of aggregates, and the development of disease remains ill understood.

The protein αS is constantly secreted into the extracellular space by αS expressing cells in the brain [4]. More importantly, extracellular αS monomers and aggregates can be taken up by other cells [5–8]. This uptake possibly contributes to spreading of αS aggregation throughout the nervous system [9, 10]. In healthy cells αS function is associated with synaptic activity [11, 12] possibly by regulating the synaptic vesicle pool [13–15] via reclustering of vesicles after endocytosis [16–18]. In αS knockout mice, however, no clear impairment of basic brain functions or neuronal survival could be observed [19]. Therefore Chandra et al hypothesized that αS may have a function in preventing the accumulation of non-native, potentially toxic molecules during the continuous operation of axon terminals [20], like suggested for another abundant protein, cysteine string protein alpha (CSPα) [21, 22].

It is estimated, on the basis of radioactive immuno blots and quantitative mass spectrometry, that the intracellular αS concentration accounts for ~0.5% of total brain protein [14, 23, 24]. With a protein concentration of about 100–200 mg/ml [25], this corresponds to a physiological αS concentration of 35–70 μM in the human brain and ~40μM in the rat brain [26]. For Ubiquitin, cytoskeletal actin, and another Parkinson associated protein, UCH-L1, similar concentrations can be found in the cortex [23, 24, 27]. Mutations in the SNCA gene encoding for αS are directly associated with the development of synucleinopathies and decrease the disease onset [27, 28]. On average, a 1.6 times higher αS concentration is found in neurons of the substantia nigra of elderly people than in young adults as observed by optical densitometry of immune stained tissue slices [29]. A recent post mortem study using western blot showed a fivefold increase in αS concentration in the substantia nigra of brains of MSA patients and a twofold increase in the striatum of MSA and PD patients, as compared to post mortem brains not diagnosed as MSA or PD [30]. Thus, under pathological conditions, local intracellular αS concentrations well above 100 μM are possible, but large differences are found between different cell types and part of the increase can be attributed to presence of αS in inclusion bodies [31]. Much lower concentrations have also been reported in other cell types [32]. Although αS concentrations in cerebrospinal fluid are on average in the nM range (1–300 pg/μl) [33], the extracellular concentration may locally rise much higher, e.g. in case of cell rupture or sudden cell death as a result of mild brain trauma [34, 35]. The extracellular concentration in such cases is unknown, but it may locally increase towards the intracellular concentration. This extracellular αS is possibly removed due to re-uptake by glial cells and neurons [36, 37], transport from CSF to the blood serum [38, 39] and degradation by extracellular matrix proteases [40–43]. Earlier work showed that neurons take up excessive αS, leading to the formation of Lewy body like inclusions [37]. The physiological goal of this process remains unclear. Recent work suggests that the formation of LBs occurs simultaneously with synaptic impairment and neuron death, but the exact mechanisms remain unknown [11, 12]. Neuronal uptake of excessive αS has been suggested to be a cellular defense mechanism [44], or to occur as a consequence of the physicochemical conditions in the cell [45], which may in evolutionary perspective be strongly associated. Either way, biology may have found ways to prevent buildup of excessive extracellular αS. Therefore, we hypothesize that temporal excessive extracellular αS might be an initiator of αS related pathology.

We exposed cultured rat cortical networks on multi electrodes arrays (MEAs) to recombinantly expressed human αS (hαS), to investigate the possible detrimental effects of high concentration extracellular αS, e.g. conditions where the extracellular αS concentration approaches the intracellular concentration. Furthermore, we determined if and at what rate LB like inclusions are formed, and investigated the sequence of events that eventually lead to neuronal cell death.

## Materials and methods

### Recombinant proteins and reagents

The expression of human wild type αS (hαS) and the A140C mutant (hαS 140C) with a single alanine to cysteine substitution at residue 140 was performed in E. coli B121 (DE3) using a pT7 based expression system. Details on the purification procedure of recombinantly produced hαS are described elsewhere [46]. Purified protein was stored at -80°C in aliquots until further use. The hαS A140C monomers were conjugated with AlexaFluor488 maleimide following the manufacturer’s labeling protocol (Life Technologies, USA). Antibodies specific for αS, Amino acids 15–123, 123–140, and 96–140 were obtained from BD biosciences, Abcam, and Santa Cruz Biotechnology, respectively. Rabbit anti-MAP2 antibodies and all Alexa dye coupled secondary antibodies were acquired from Invitrogen.

### Aggregation of recombinant hαS

Aggregation of hαS was monitored using the amyloid binding dye Thioflavin T (ThT; excitation: 446nm, emission: 485nm). Fibrilization reactions were set up using 0, 10, 20, 50 and 100 μM αS and 20 μM ThT in R12 medium. The aggregations reactions were performed in 200 μl volume in 96 well plates with optically transparent bottoms, sealed with adhesive film in 6 fold, at 37°C under quiescent conditions and monitored using a Safire microplate reader (Tecan). The fluorescence intensity was recorded every 15 minutes for 8 days. Before each reading the samples were subjected to five seconds orbital shaking to homogenize the samples. To enable interpretation of intensity values, a control 100 μM sample in 10mM NaCl, 10mM Tris buffer was prepared and allowed to aggregate at 37°C with orbital shaking. Apart from the orbital shaking the ThT fluorescence intensity of these samples was followed in time under the same experimental conditions. Additionally, pre- and post- ThT incubation samples were loaded on native 4–12 gradient PAGE gels. As a positive control, αS was oligomerized as described elsewere [47].

#### Atomic force microscopy

Atomic force microscopy (AFM) samples were prepared by adsorbing 10 μl of undiluted R12 medium or undiluted solutions of αS incubated in R12 medium for 8 days on freshly cleaved mica for 4 minutes. These samples were subsequently gently washed twice with 100 μl Milli-Q water and dried using a gentle stream of nitrogen gas. AFM images were acquired on a Bioscope Catalyst (Bruker) in tapping mode in air using a silicon probe (NSC36, tip B; Mikromasch). All images were captured with 512x512 pixels at a scan rate of 0.5 or 2.0 Hz. The images were plane corrected using Scanning Probe Image Processor (SPIP)-6.0.13 software (Image Metrology).

#### ANS binding assay

To detect and characterize partially folded oligomeric αS species [48] a 200 μM 1-anilinonaphthalene- 8-sulfonic acid (1,8-ANS) stock solution in methanol was prepared. From the aggregation reactions 50 μl aliquots were diluted to 2 ml in 10 mM Tris-HCl, pH 7.4 and 40 μl of 1,8-ANS stock solution was added. As a reference sample 40 μl of 1,8-ANS stock solution was added to 2 ml of 10 mM Tris-HCl, pH 7.4. Fluorescence emission spectra were recorded on a Cary Eclipse fluorescence spectrophotometer (Varian) with excitation at 395 nm and emission detection from 410 to 600 nm using 5 nm slit widths.

### Primary neuron extraction and culture

The extraction and culturing of primary neurons was performed as described elsewhere [49]. In short, cells were obtained from newborn Wistar rats from an in-house breeding program using rats obtained from Harlan, Horst, the Netherlands. Rats were decapitated with no prior anesthesia, cortices were isolated, and cells were dissociated by trypsinization and trituration and subsequently cells were plated on polyethyleneimine (PEI; Acros Organics, USA)-coated culture dishes with glass bottoms or PEI-coated coverslips to ~60% density. After 2 hours, adhered cells were washed with Dulbecco’s Modified Eagles Mediun (DMEM; Invitrogen, USA) and cultured in 900 μl, serum-free R12 medium [50] at 37°C with 5% CO_2_. Medium was refreshed twice a week by replacing one third of the medium by the same amount of fresh medium. After three weeks cultures were mostly confluent, and were considered mature.

For electrophysiological experiments, cells were plated on multi electrode arrays (MEA; Multi Channel Systems, Reutlingen, Germany), containing 60 titanium nitride electrodes (30 μm diameter and 200 μm pitch). Cells were plated on MEAs at a final density of approximately 2500 cells/mm^2^. For experiments 50 or 100 μM αS was added to mature neural networks. As a control the small stable moderately non-reactive protein bovine serum albumin (BSA; Sigma-Aldrich; 100 μM) was used. For electrophysiological recordings, we firmly sealed the culture chambers with watertight but CO_2_ and O_2_ permeable foil (MCS; ALA scientific), and placed the cultures in a measurement setup outside the incubator while maintaining temperature, pCO_2_, and humidity. Experiments lasted five to seven days. During experiments the culture medium was refreshed once, after four days. Fresh medium did not contain new αS or BSA. All experiments were done at least 20 days after plating. Recordings began after an accommodation period of 20 minutes. All procedures involving animals were conducted according to Dutch and European laws and guidelines, and approved by the Dutch Animal Use Committee (DEC).

### Immunocytochemistry

Cell samples were washed with PBS and fixed in 3.7% paraformaldehyde/PBS solution. For immunolabeling, cells were permeabilized with 0.3% Triton X-100 and 0.1% BSA in PBS. Autofluorescence was quenched with 50 mM NH_4_Cl in PBS. Primary antibodies were applied in 16% goat serum, 0.3% Triton X-100, 0.3 M NaCl in PBS, and incubated overnight. Subsequently, cells were washed three times with 0.3% Triton X-100 and 0.1% BSA in PBS at room temperature. Secondary antibodies were applied in same buffer and incubated for one hour. For ThioflavinS (ThS) or phalloidinalexa647 staining, fixed cells were incubated with 0.05% ThS or 70 nM phalloidinalexa647 in PBS for 15 minutes. Nuclear counterstaining was performed by incubation in 300 nM 4',6-diamidino-2-phenylindole (DAPI) in PBS for 10 minutes. After washing with PBS, samples were mounted with mounting medium (Ibidi, Germany).

### Confocal microscopy

Confocal laser scanning microscopy images were obtained on a Zeiss LSM510 Confocal microscope with a 63x oil immersion objective (NA = 1.4, Zeiss, Germany). Images were taken successively, and were analyzed with the ZEN software 2009 (Zeiss, Germany).

### Quantifying the number of αS deposits

To quantify the number of αS deposits, images of three random regions (150 x 150 μm) of samples immunolabeled for αS were obtained at different time points with confocal microscopy. The fluorescence intensity from the αS deposits is typically very high. In the image analysis procedure we therefore set a threshold above which only αS deposits were visible. The same threshold was applied to all images. The αS deposits and cells (DAPI) per image were counted and averaged.

### Immunoblot analysis

Cells were incubated in buffer [4x Laemmli buffer (8% SDS, 240 mM Tris-Cl, pH 6.8), 100 mM DTT] for 15 minutes at room temperature and transferred to Eppendorf tubes. The samples were boiled for five minutes and centrifuged for two minutes at 12000 rcf (IEC MicroMAX tabletop centrifuge). Subsequently, the proteins in the samples were separated on a 12% SDS-PAGE gel. The resulting gel was imaged (excitation.: 488 nm, emission.: 510–600 nm) on a gel imaging system (Gel DocTM, Bio-Rad, USA).

### Metabolic activity assay/ live dead assay

Cells seeded in 24-well plates (Greiner Bio-One GmbH, Germany) were incubated with 100 μM hαS monomers or 100 μM rotenone for one, five, or seven days. Rotenone was used as positive control for toxicity. Cells were incubated with 0.5 mg/ml 3-(4,5-dimethylthiazol-2-yl)-2,5-diphenyltetrazolium bromide (MTT) in medium for four hours at 37°C in an atmosphere containing 5% CO_2_. The medium was discarded carefully and cells were solubilized in DMSO. After cell solubilization, the metabolic activity was quantified in a multiwell plate reader by measuring the absorbance at 540 nm (Tecan Ltd, Switzerland). The background absorbance was determined at 690 nm and subtracted from the MTT signal. The MTT data of treated groups were normalized to a control at the same time point. In short, cells were plated on glass cover slips, washed with cold PBS and incubated with 100 μl/ml solution of propidium iodide (PI) and/or annexin V (Invitrogen, USA) for 15 minutes at room temperature. Subsequently, cells were washed with binding buffer. Images were obtained using an inverted fluorescence microscope (EVOS, AMG, USA) with a 20x objective (N.A. = 0.4, EVOS, USA).

### Activity recording and wave shape analysis

Action potentials (spikes) on MEAs were measured extracellularly, generally with a negative potential [51]. We calculated the array wide firing rate (AWFR) as the summed activity of all electrodes in one hour time bins. In continuous recordings, 12 consecutive bins were averaged to monitor AWFR at a temporal resolution of two samples/day. In discontinuous recordings (particularly control experiments with BSA), all one hour bins with an AWFR value that fell within a 48h interval were averaged per culture, and then averaged across cultures. AWFR generally increases during the first three weeks after plating. After that, from week three to week seven, predominantly minor fluctuations are observed and a fairly stable mean firing rate on a time scale of hours to days can be observed [52]. Firing patterns usually contained periods of seemingly uncorrelated firing, alternated by periods of highly synchronized firing at many electrodes (network bursts). Data were recorded from all electrodes at a sample rate of 16 kHz, using a custom Labview (National Instruments, Austin (Tx), USA) program. Potential spikes were stored whenever the signal exceeded a predefined threshold of 5.5 times the estimated noise level (which was continuously updated). For each spike, the program stored the time stamp, the recording electrode and 6 ms of the spike waveform (from 2 ms before to 4 ms after the threshold crossing).

Stored wave shapes were used for off-line artifact detection following the procedure described in [53]. If αS affects neuronal viability, or, more generally, the resting membrane potential [54], this is also reflected in a change in the action potential, which can be detected in extracellular potential recordings [55]. In MEA recordings, electrodes may pick up signals from more than one neuron. For this analysis, however, we selected electrodes that recorded activity from a single neuron, following the approach introduced in [56]. In short, a measure is calculated for the variability of action potential shapes at each electrode. Electrodes with sufficiently low variability during baseline were considered as single neuron electrodes. Wave shapes were averaged per electrode in 1 h time bins. In each bin we calculated the relative amplitude (normalized to baseline) and the correlation coefficient between the action potential shape of that hour and those of all preceding hours. All activity of individual cultures was normalized to the baseline activity before averaging across cultures. Finally, for each neuron, the time of last activity was determined. Neurons with a correlation coefficient of more than 0.9 and a relative amplitude of more than 0.75 at the time of last activity, were considered to have unchanged action potential shapes.

In addition to spontaneous activity recordings, some cultures were electrically stimulated during 25 minutes per two hours. In these experiments, all data recorded from the start of the stimulation period until 10 minutes after this period were excluded for the analysis of spontaneous activity, wave shape, and synchronicity (see below).

### Burst synchronization analysis

Synchronized network bursts depend on synaptic transmission, and as such provide a suitable indicator of synaptic functioning. To analyze the relative occurrence of network bursts we determined a burstiness index (BI) [57]. In short, five minute recordings were divided into 300 (1s) time bins and the total number of spikes in each bin, summed across all electrodes, was counted. The number of spikes in the 45 bins (15% of all bins within the 300 s period) containing the largest spike counts (f15) was expressed as a fraction of the total amount of spikes in that 300 s period. Should most of the spikes occur in bursts, f15 will be close to 1; tonic firing should lead to f15 ≈ 0.15. We then defined a BI such that BI = (f15-0.15)/0.85, such that BI is normalized between 0 (no bursts) and 1 (burst dominated). Five minute periods with less than 600 spikes were excluded from this analysis.

### Stimulation experiments

Diminishing neural activity after αS addition may reflect decreased neuronal excitability or impeded synaptic transmission. To differentiate between these two possible causes of reduced activity, cultures were electrically stimulated (bi-phasic square pulses, 200 μs per phase, 16–32 μA, one stimulus pulse per 10 seconds during 25 minutes per two hours). [58]. Electrical stimulation has been shown to induce a response in two phases. The first phase results predominantly from direct stimulation of neurons in the near proximity of the stimulating electrode and (antidromic) activation of axons in this area [59, 60]. The second phase, called network or synaptically mediated response, results from synaptically propagated signals neurons that fired in the first phase to connected neurons, thus creating a wave of activity throughout the network. The first phase (early response) is relatively fast, it typically terminates within 5–15 ms, and besides directly induced action potentials it may contain some synaptically induced spikes [61]. The synaptically mediated response, typically 15–300 ms after the stimulus, can be completely abolished by Glutamatergic receptor antagonists (2R)-amino-5-phosphonovaleric acid (APV) and 6-cyano-7-nitroquinoxaline-2,3-dione (CNQX) [62]. All cultures were stimulated using three pre-selected electrodes that were able to trigger a good network response. The lowest amplitude that was able to induce a network response was determined, usually in the range of 16–32 μA. The stimulation protocol consisted of pseudo random stimulation of the three selected electrodes at 10 seconds intervals. Each electrode was stimulated 50 times, resulting in a total stimulation period of 25 minutes. The stimulation period was repeated every two hours. Only stimulation electrodes that showed an averaged, (combined direct and late) response of at least 60 action potentials per stimulation during baseline measurements were included in this analysis.

## Results

### Recombinant hαS does not aggregate into amyloid fibrils in medium

**Fig 1:**
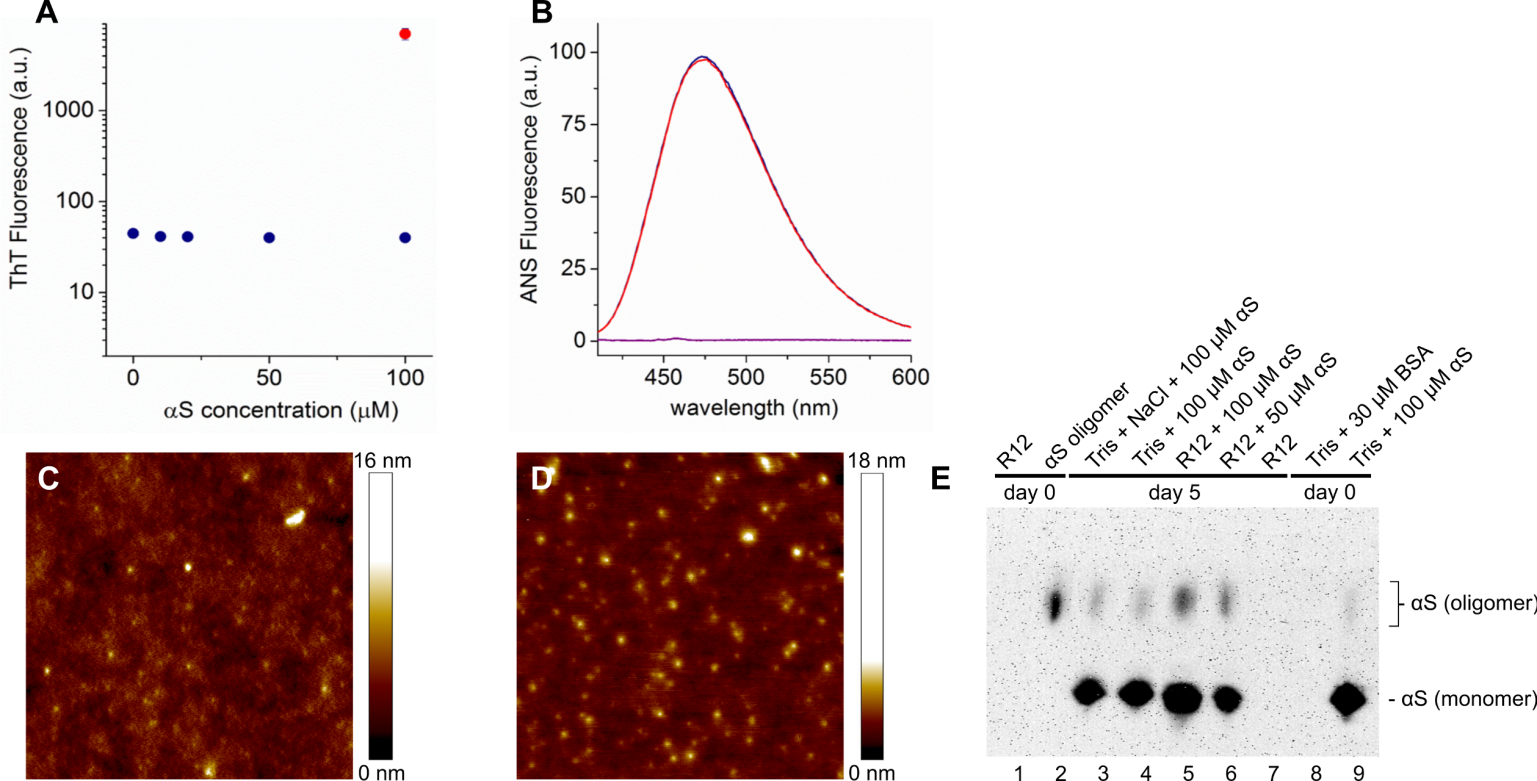
hαS does not aggregate into amyloid fibrils in R12 medium. A) Fluorescence intensity of the amyloid binding dye ThT as a function of the hαS concentration after incubation in R12 medium for eight days at 37°C (blue) and hαS fibril control sample (red). B) Fluorescence spectra of 1,8-ANS incubated in R12 medium for eight days at 37°C in the presence of 100 μM hαS (red) and without hαS (blue). As a reference the fluorescence spectrum of 1,8-ANS in 10 mM Tris-HCl, pH 7.4 is shown (purple). C) Representative atomic force microscopy (AFM) height image of a 50 μM hαS sample after eight days of incubation in R12 medium indicating that the samples contained small structures. D) In AFM images of the deposited R12 medium with no added hαS similar structures were found as those observed in C). E) Native western blot showing hαS after incubation in 10mM Tris buffer or R12 medium for 0 and 5 days at 37°C.

After eight days of incubation of hαS in medium the ThT fluorescence intensity of this solution was comparable to the ThT intensity observed for the buffer control sample. Moreover, the ThT intensity did not increase with increasing hαS concentration (**Fig 1A**). The ThT intensity of medium containing hαS was two orders of magnitude lower than that of control samples containing αS amyloid fibrils, suggesting that no amyloid fibrils were formed. The absence of amyloid fibrils in medium was confirmed in AFM images. However, the images show small structures with a height of approximately 10 nm (**Fig 1C**). Although this height agrees with the diameter observed for αS oligomers [47, 63], similar structures are found for R12 medium deposited on mica (**Fig 1D**). We did not observe any difference in ANS fluorescence between R12 medium with or without 100 μM αS (**Fig 1B**). Western blots of hαS in R12 medium, made after five days of incubation, showed very low amounts of oligomeric αS (**Fig 1E**). Control blots indicated that these oligomeric species were already present before R12 incubation. We therefore conclude that in the experimental time frame no fibrils are formed but the solution does contain small amounts of oligomeric αS species.

### Primary neurons contain endogenous αS and take up extracellular αS

**Fig 2:**
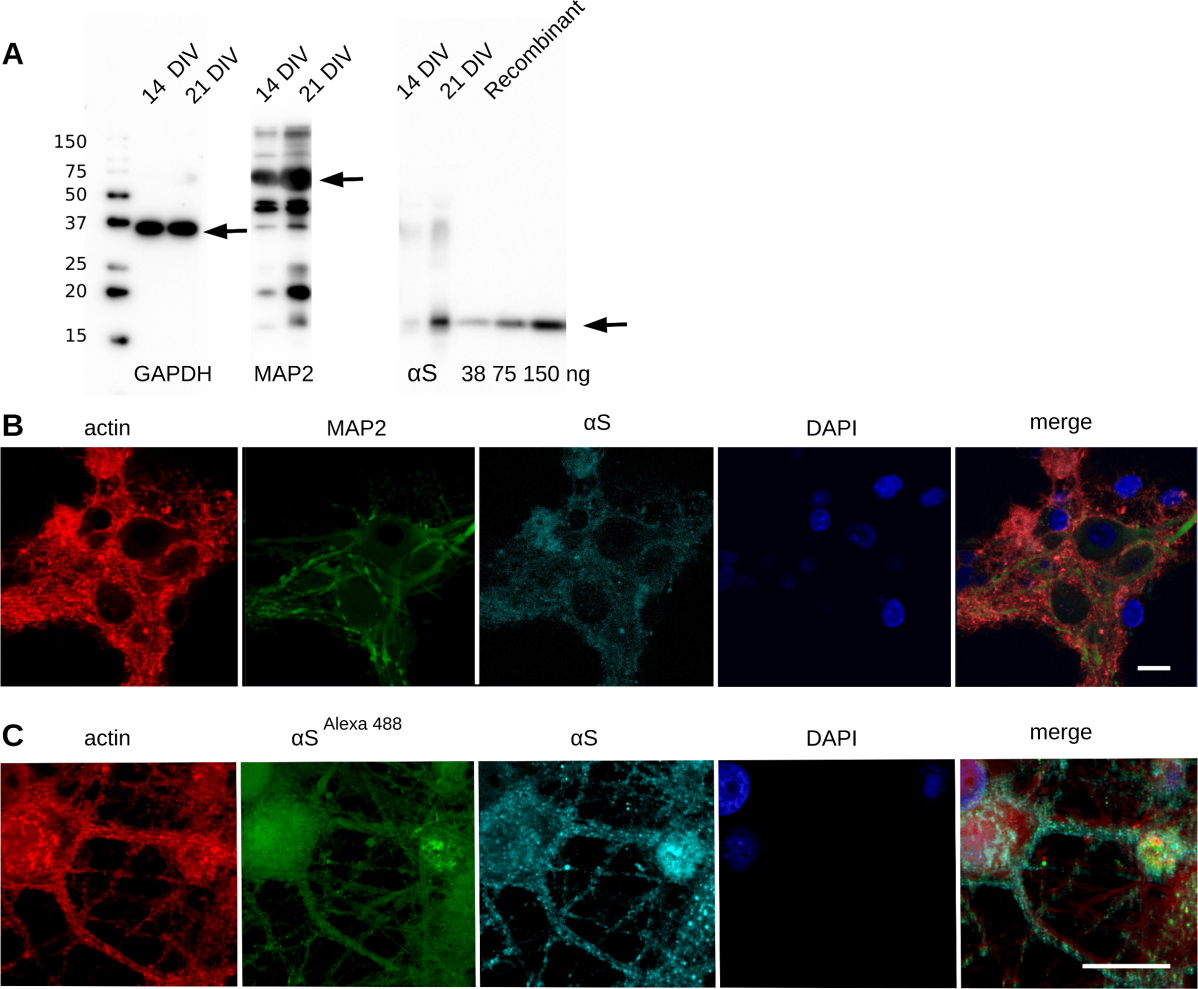
Primary neuronal cells express endogenous rαS and neuronal marker MAP2. A) Immunoblot of cell lysate of primary neuronal cells after 14 days shows immuno-reactivity for MAP2 and αS. B) Cells were fixed and fluorescently labeled; red: phalloidin ^alexa647^, cyan: αS ^alexa594^, green: anti-MAP2 ^alexa488^, blue: DAPI; confocal microscopy; scale bar 10 μm. C) Cells were treated with 100 μM labeled hαS ^alexa488^ and unlabeled hαS monomers (ratio 1:3) for six hours and processed as in B; red: phalloidin ^alexa647^, green: exogenous hαS ^alexa488^, cyan: anti-αS ^alexa594^ (endogenous rαS and exogenous hαS), blue: DAPI; confocal microscopy; scale bar 5 μm.

We tested eight neuronal cultures for expression of rat αS (rαS) by immunoblot analysis at different time points. Two weeks after plating we observed expression of rαS. The molecular weight of the endogenous rαS is comparable to that of recombinant expressed human αS (hαS). After three weeks we observed increased expression of both endogenous rαS and the neuron specific marker MAP2, while the expression of the housekeeping enzyme GAPDH stayed constant (**Fig 2A**), indicating that neurons matured during that time. Quantification of the rαS expression using the blot and taking recombinant hαS as a standard (**Fig 2A lanes 8–10**) resulted in an estimate for the total concentration of rαS of 20–50 μM αS at 21 days in vitro (DIV). This is comparable to the rαS concentration in rats reported by others [26]. Immunostaining of the cells showed that rαS was expressed only in MAP2 positive neurons/cells (**Fig 2B**) and homogeneously distributed throughout the cell.

To visualize the uptake of extracellular hαS, fluorescently labeled monomeric hαS (hαS alexa488) was added to the culture medium. With confocal microscopy, we observed significant uptake of hαS alexaA488 by cells within six hours after addition, in both cytoplasm and nucleus (**Fig 2C**).

### Exogenously added hαS hardly affects cell viability and metabolic activity

Increased amounts of endogenous αS can be toxic to cells [11, 64–72]. To investigate whether this also applies to exogenously added hαS, we evaluated the cytotoxic effect of externally applied hαS on the cultures of primary rat neurons. Exposure to hαS did not affect cell substrate adhesion as we did not observe increased cell detachment, compared to the control group (data not shown). Neither the live-dead marker, propidium iodide, nor the apoptotic marker Annexin V, showed a significant increase in positive cells for hαS exposed samples compared to control cells (hαS 1.3 ± 1.4% compared to control 1.8 ± 2.0%, P = 0.34 (t-test) and hαS 7.2 ± 7.2% compared to control 3.8 ± 3.4%, P = 0.51, respectively) after seven days of treatment.

Since we did not detect an effect on cell viability, we next evaluated whether exogenously added hαS has an effect on the metabolic activity as measured in a MTT assay. Metabolic rates tended to increase immediately after exposure to hαS to 131.6 ± 8.4%, compared to control samples (P = 0.25; t-test). After seven days metabolic rates had returned to control values (102.1 ±7.7%). In the presence of rotenone, a significant decrease of metabolic activity was observed after addition (47.2 ±2.0%; data not shown in Figure).

### Exogenous hαS promotes intracellular αS deposit formation

**Fig 3:**
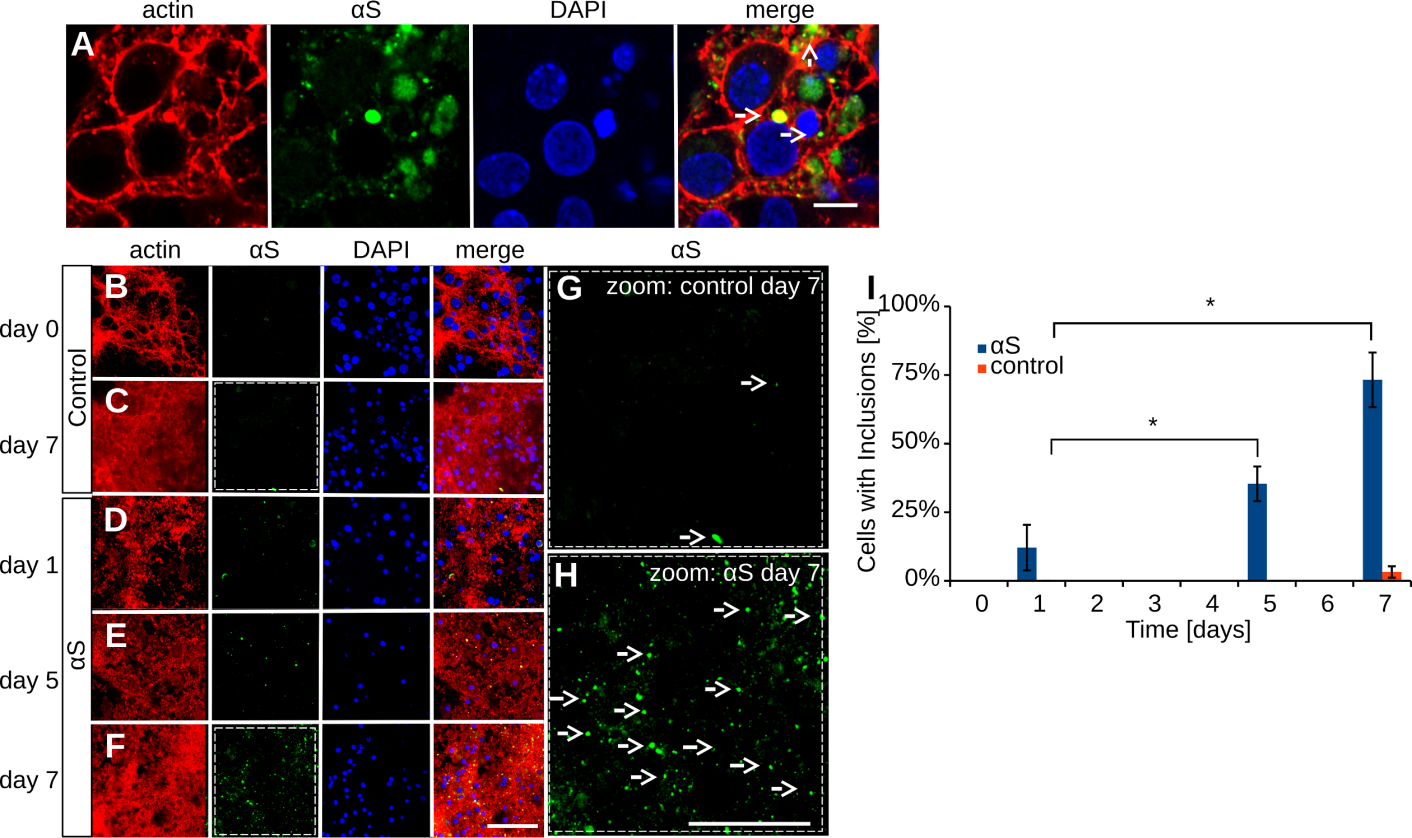
Exogenous hαS induces αS deposit formation in primary cortical cells. Cultured cortical cells were exposed to 100 μM hαS monomers for one, five and seven days. Cells were fluorescently labeled with actin^phalloidin647^: red and anti-αS ^alexa594^: green; confocal microscopy; A) After hαS exposure, αS deposits were observed in the cells. Note the actin positive staining of deposit (middle arrow), confirming the intracellular location. B-C) αS deposits formed in control cells. D-F) αS deposits formed in cells exposed to exogenous hαS. G-H) Magnification of αS immunolabeled cell cultures at day seven. I) The number of αS deposits per cell; n = 3 for all groups; paired student’s t-test: * indicates P<0.05; scale bar 5 μm (A), 50 μm (B-F) and 10 μm (G-H); αS deposits are indicated by white arrows.

To assess if the high extracellular hαS concentration induced the formation of αS deposits, cells treated with hαS were immunolabeled for αS and analyzed by confocal microscopy. **Fig 3A** shows that αS deposits larger than 500 nm were formed within seven days. Occasionally deposits were also actin positive, indicating their intracellular location, as visible in **Fig 3A**, middle white arrow. A more detailed characterization of αS deposits can be found in Raiss et al. 2016 (Sci Rep). The number of αS deposits increased in time (**Fig 3B–3H**). Whereas only few αS deposits were found after one day of αS exposure, about a third of the cells contained an αS deposit by day five, and 75% of all cells contained a deposit by day seven (**Fig 3I**).

### Exogenous hαS alters neuronal activity and burst synchronization but leaves the action potential amplitude and shape unchanged

**Fig 4:**
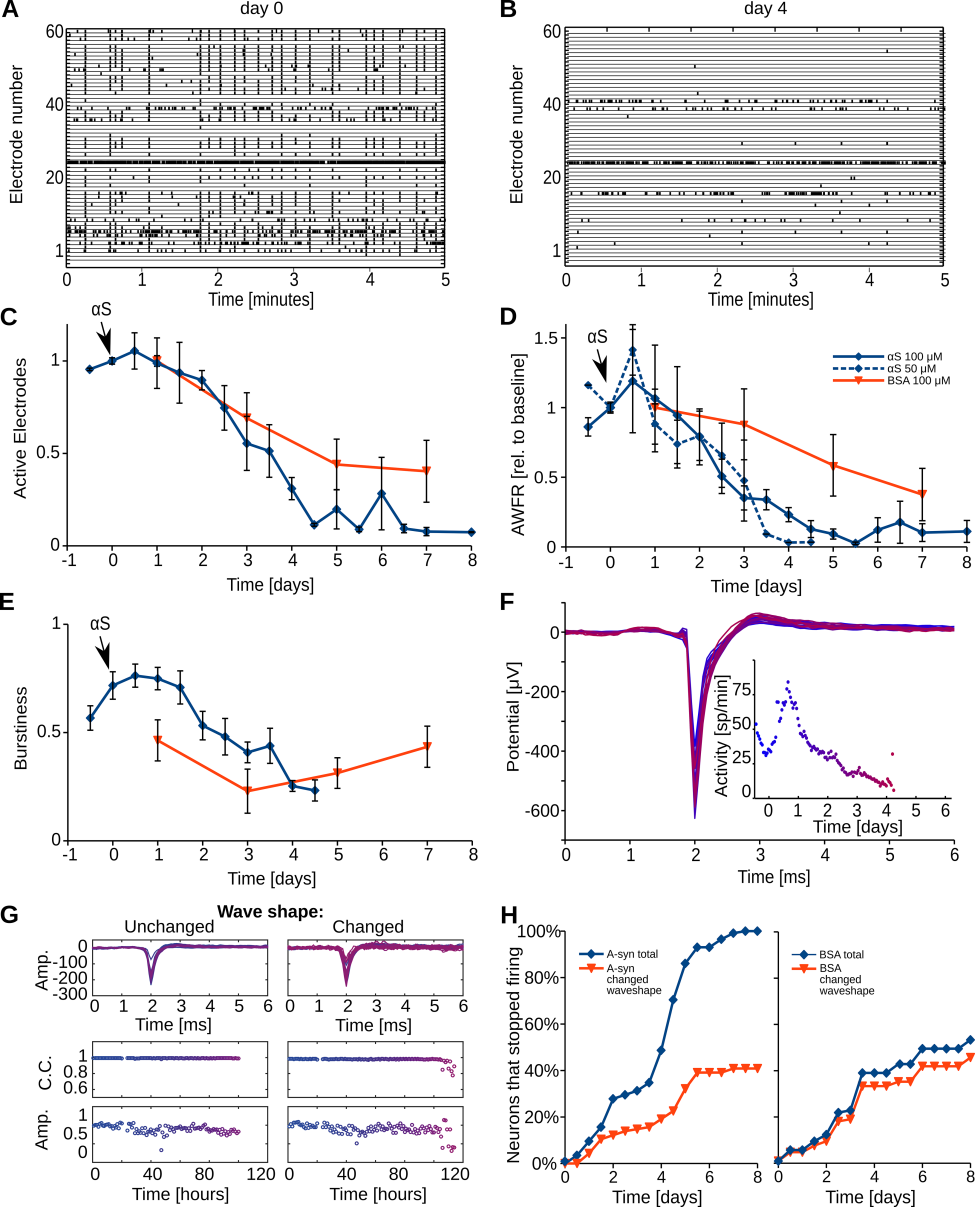
Exogenous hαS decreases neuronal activity and synchronicity of neuronal action potential firing pattern. Rat primary cortical cells were cultured for three weeks and subsequently exposed to either 100 μM hαS or BSA. Neuronal activity was recorded using microelectrode arrays. A) An example of baseline neuronal activity. On the vertical axis, all 60 electrodes are indicated and each tick in the corresponding row represents an action potential. During baseline recordings, started 30 minutes before hαS administration, on average around 50 of the 60 electrodes were active and all activity patterns included network bursts (synchronous firing at all or most electrodes, visualized as ‘vertical columns’). B) Recording of the same network four days post hαS administration. C-D) The average number of active electrodes and Array wide firing rate (AWFR; the summed activity of all electrodes in one hour time bins, divided by the bin length) were registered for eight days from eight αS treated cultures (blue) and three BSA (red) [95] treated cultures. E) Neuronal synchronicity slightly increased for two days and then decreased, as indicated by the development of the burstiness index. BSA treated cultures initially showed a similar pattern but here the burstiness recovered after four days F) The action potential shapes and the activity (dots; inset) of a representative neuron at different timepoints (color shift; day 0 = blue, day 7 = red) after addition of αS. G) Examples of neurons with unchanged and changed wave shapes. Top row, overlay of action potentials from every hour color coded as in F. Middle row, correlation coefficients at every hour. Bottom row, action potential amplitudes at every hour. Note the drop in correlation coefficient and amplitude in the last hours in right versus left panel. H) Cumulative histogram showing the fraction of all neurons, with reproducible baseline action potential shape (n = 115), that became inactive in time. Blue indicates all neurons, red shows the fraction of neurons for which the action potential shape changed before becoming inactive. C-E: Error bars represent SEM; n = 8 independent experiments.

In eight cultures, we recorded electrophysiological activity, and we investigated the effect of the added recombinant hαS. Initially, all cultures were active (9.9 ± 2.3 (SEM) spikes per second) and their activity patterns included periods of highly synchronized action potential firing, i.e. all cultures regularly showed network bursts. The array wide firing rate showed an initial increase, directly after administration of hαS, which lasted from a few hours up to a full day. The magnitude of the increase varied per experiment as reflected by the relatively large error bars (**Fig 4D**). After the initial increase, the AWFR decreased and dropped below baseline values within approximately 24 hours. The firing rate decreased significantly until day five, when hardly any activity could be recorded (one-way ANOVA: p<0.01, **Fig 4D** and compare **Fig 4A and 4B**). Whereas initially action potentials were recorded at most electrodes, with time an increasing number of electrodes became silent particularly beyond day three (p<0.001, **Fig 4C**). The burstiness index remained stable for the first two days and then decreased significantly (p<0.001, (**Fig 4E**). After five days the activity was too low to calculate the burstiness index. In four control cultures treated with 100 μM BSA, only the number of active electrodes decreased with time (one-way ANOVA: p<0.02), but to a significantly lesser extent than in hαS treated cultures (two-way ANOVA: p<0.05). AWFR and burstiness were not significantly affected (p = 0.18 and p = 0.29, respectively, **Fig 4C and 4E**).

We next analyzed the temporal development of the shapes of the action potentials, at those electrodes that recorded single unit activity. Each presented action potential is the average of all action potentials recorded at a particular electrode within a one-hour period. Superposition of all resulting averaged action potentials of a representative electrode clearly shows that shape and amplitude of the averaged action potential remained constant throughout the experiment (**Fig 4F**). This constant shape of the action potential over time was observed at the majority of the selected electrodes. **Fig 4H** shows that a significant portion (60%) of neurons had unchanged action potential shapes when they stopped firing. (**Fig 4G and 4H**). In contrast to the 100% silencing observed in αS treated cultures, only 50% of the neurons had stopped firing after eight days in cultures treated with BSA. Of those neurons only 5% showed unchanged action potentials. Remarkably, most action potential shapes, if changed at all, did not change gradually over time, but rather suddenly during the last hours before they stopped firing, as shown in **Fig 4G right panel**.

### Synaptic transmission is hampered in hαS exposed neurons

**Fig 5:**
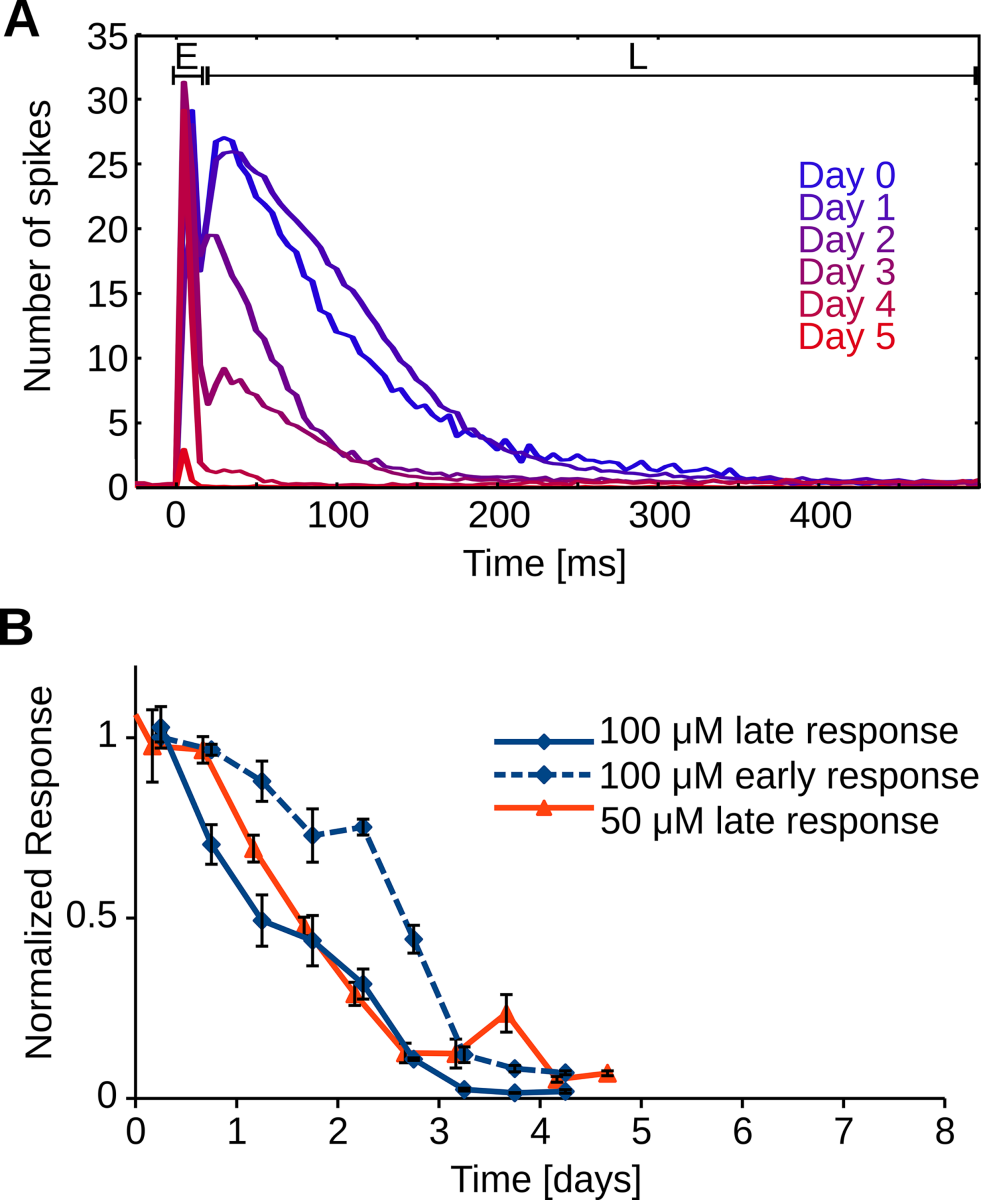
hαS interferes with intercellular communication. Networks of primary cortical rat neurons are electrically stimulated before (day 0) and after administration of 100 μM hαS. A) The first 15 milliseconds after stimulation are dominated by action potentials that were directly triggered by the stimulus pulse (early response; E). These early action potentials are then synaptically propagated through the network and neurons at other electrodes respond up to 300 milliseconds after the stimulation (late response; L). Time progress is visualized by shifting color from blue (day 0) to red (day 5), every line represents the averaged result of 12 stimulation periods (600 stimulations). B) Average development of early and late response after addition of 50 or 100 μM αS, normalized to baseline values. C) Average activity for neural networks treated with 50 μM αS. n = 9 electrodes in three different preparations for each condition.

To assess the quality of synaptic transmission, early (5–15 ms latencies) and late responses (15–300 ms) to electrical stimulation were measured and normalized to baseline responses (before the addition of hαS, **Fig 5A**). Late responses started to decrease almost directly after hαS administration whereas early responses remained around baseline for two days. The late response continuously decreased for four days until we were no longer able to measure any late responses (**Fig 5B**). A similar temporal evolution was observed when αS was administered at 50μM for both late response (two-way ANOVA: p = 0.17 **Fig 5B**) and AWFR (two-way ANOVA: p = 0.28 **Figs 5C and 4D**). Two control cultures treated with BSA were electrically stimulated, late responses remained above 80% of their baseline response for at least 40 and 100 hrs, respectively, after which the recordings were stopped (data not shown).

## Discussion

### Cascade of events

**Fig 6:**
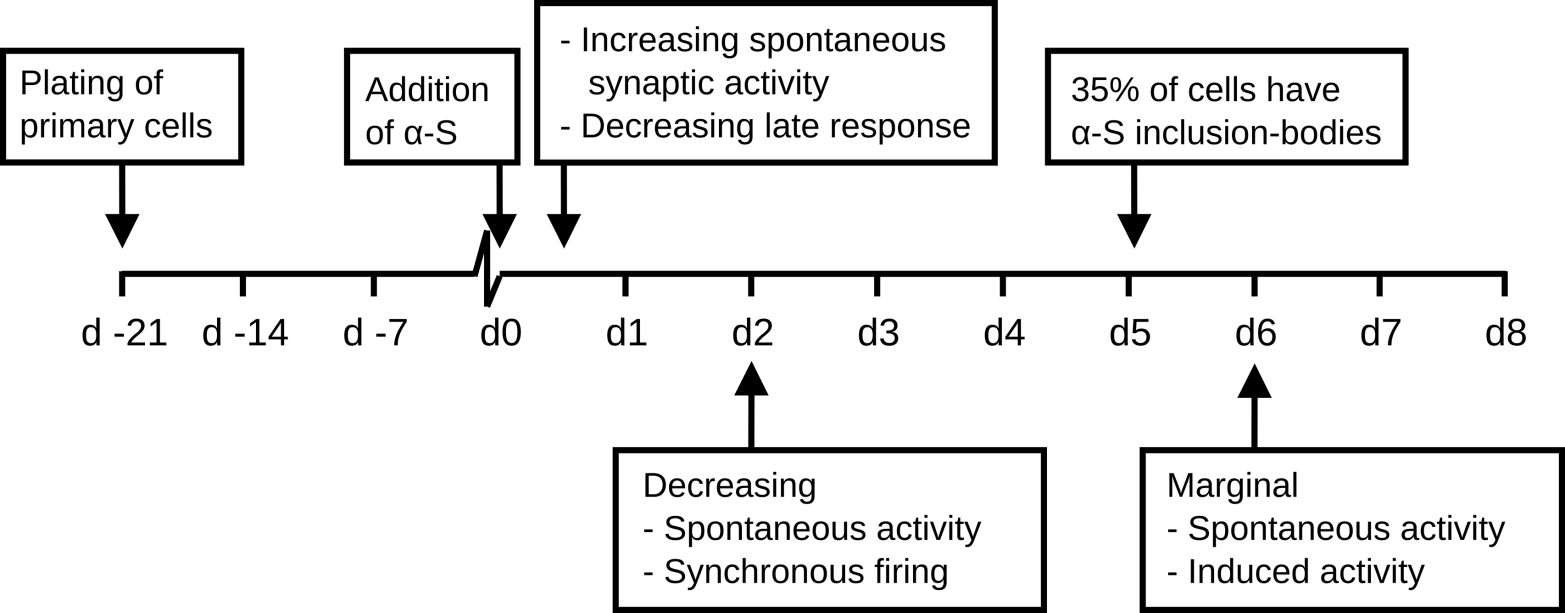
Timeline of events. Three weeks after plating, when neural networks were considered mature, cortical cell cultures were treated with 100 μM αS. Within the first day after αS addition spontaneous synaptic activity rose while electrically induced network response started to decrease. Within two days after addition, spontaneous activity and the number of electrodes on which activity was recorded dropped below baseline. At day five, on average, about one third of the cells had an inclusion body. By day six hardly any spontaneous activity could be recorded and electrical stimulation was no longer able to evoke a network response.

Within six hours after the administration of monomeric hαS to mature neural networks derived from rat cortical cells, we observed uptake of fluorescently labeled hαS. Although possible mechanisms on how extracellular proteins can enter the cytoplasm have been proposed, the exact mechanism remains unclear [73]. Between 24 to 48 hours after hαS addition, network excitability started to decrease, as demonstrated by the vanishing synaptically mediated phase of the responses to electrical stimulation. Network excitability may be defined as the average network response to a small stimulus, and thus covers neuronal excitability and synaptic efficacy. Network synchronicity (burstiness) also dropped, confirming the decreasing network excitability. Two days after hαS administration, network excitability dropped to almost zero, but individual neurons were still intact and functioning as demonstrated by the remaining high number of active electrodes, of which action potential shapes and amplitudes were unaffected. At that time, the increase in intracellular hαS concentration resulting from uptake of monomeric protein had resulted in the formation of αS deposits in only a few cells. It generally took five days before one third of the cells contained at least one deposit. Electrophysiological changes, reflected by a decrease in the number of active electrodes, were observed much earlier, between three and four days after αS administration. This decrease in the number of active electrodes might suggest that cells became fully dysfunctional and died. However, cell death and metabolic assays did not support this, leaving the conclusion that the number of active electrodes probably decreased because of decreased network excitability (**Fig 4D**). Thus, network excitability significantly dropped long before LB like deposits became visible. The time line of the events observed in this study are summarized in **Fig 6.** It seems unlikely that the effect on network excitability and the consequent drop in network activity result from the presence of large intracellular αS deposits. The relatively late formation of inclusion bodies is in agreement with a study by Volpicelli et al [11], who saw first inclusion bodies four days post addition of preformed fibrils.

### Interference with network functioning

The decrease in activity might result from neuronal dysfunction, increased inhibition, or from massive synaptic failure. Although a decrease in network activity and failure of isolated synapses in neuronal cultures treated with αS have been reported before [11, 12] we, for the first time, show directly and in a large number of neurons, that impairment of network excitability after addition of αS occurs *before* neuronal degradation. The fact that we were able to stimulate cells directly while spontaneous activity was reduced significantly and action potential shapes and amplitudes remained unaltered, supports the view that reduced activity is not due to neuronal dysfunction. Interference of αS with specific non-synaptic ion channels in the neuronal membrane [74] would alter wave shapes, as would other mechanisms that involve changes in membrane conductance [75–79] or cell death [80]. Alternatively, decreasing activity might be caused by activation of inhibitory receptors. If αS acts as a GABA agonist, we would expect an immediate drop in neural activity upon αS administration. During the first 24 hours, however, the total firing rate increased, while the network response to electrical stimulation already decreased. Therefore, it is unlikely that the eventual decrease in activity was due to increased inhibition. Thus, we conclude that the activity decrease is most likely caused by massive synaptic failure.

### Interference with synaptic functioning

Failure of synaptic transmission can take place at many different locations within the signaling cascade. It has been reported that extracellular αS can block post-synaptic receptors directly [81, 82]. At the cytosolic side of the post-synaptic membrane intracellular αS has been reported to enhance internalization of NMDA receptors within 60 minutes [83]. One might speculate that extracellular αS possibly sequesters neurotransmitter within the synaptic cleft, but experimental evidence to support this view is lacking. Monomeric αS or small αS oligomers in the medium might be responsible for such a mechanism, but it is highly unlikely that the intracellular αS deposits are involved.

Alternatively, the excess αS or oligomeric αS aggregates may interfere at the presynaptic side, e.g. in synaptic vesicle trafficking [16, 17] or the synaptic vesicle cycle [18]. Although oligomers may play a role in these mechanisms [63], it is improbable that large αS deposits are responsible; synaptic failure occurs well before large scale cell death. Our analysis confirms that synaptic dysfunction is responsible for synaptic failure, but our data does not allow further differentiation between the above mentioned pre- or post-synaptic mechanisms. Characterization of network activity in the presence of BSA showed that synaptic failure is not a generic effect of surplus protein.

### Initial activity increase

The underlying mechanism of the unexpected but robust initial activity increase remains unclear. αS oligomers have been shown to increase the frequency of synaptic transmission due to their pore forming properties [84]. Pro-inflammatory cytokines have been reported also to lead to increased neuronal activity and may decrease firing synchronicity [85–88]. Although we have not characterized our cultures for the presence of microglia [89, 90], this may have caused the observed activity increase. Interesting in this respect is that we do see increased, usually uncorrelated activity as well in early stages of bacterial infection of cultures (personal observation GH & JlF).

### Concentration, aggregation and timespan of changes

Recent studies attributed different effects to specific different αS fibril species [12, 91]. In other studies the time required to adjust firing patterns of neurons in response to the addition of `fibril species was longer [11, 12] than the timespan of activity changes seen in response to the addition of extracellular protein observed here. This slower response may reflect the lower αS concentration used in those studies, up to 1 μM in extracellular assemblies. To mimic the maximal possible effect, we used 50–100 μM, a concentration equivalent to intracellular αS concentrations observed in rat neurons (Fig 2A) and reported in PD and LBD brains [14, 29, 30].

We show that an increased extracellular αS concentration may induce pathology. Though it does not immediately result in cell death, neurons become isolated and network activity drops dramatically. The rapid effect of exogenous αS on the synaptically mediated phase of stimulus responses (within one day), suggests that it can be assigned to the presence of monomers or small oligomers.

### Toxicity

The addition of 100 μM hαS monomers had no noteworthy effect on cell viability or metabolic activity on the time scales studied. In agreement with [92] the cytotoxic effects of αS exposure are only small in primary cultures during the first weeks after exposure. The absence of a severe cytotoxic effect (as in SH-SY5Y [37]) might be due to the presence of glial cells, which generally fulfill a supporting function in neuronal networks. Suppressed synaptic functioning, however, generally leads to decreased neuronal activity. Reliable synaptic transmission is essential for regular calcium entry in postsynaptic neurons, which is needed for neuronal survival [93, 94] and the remaining activity may be insufficient for long-term neuronal survival. It remains to be seen whether this discrepancy between cell death and loss of synaptic transmission can also be observed in neurons derived from other parts of the nervous system and whether the same mechanism is present and crucial in the etiology of synucleopathies in patients.

## Conclusion

Combined, these data support the idea that synaptic failure is the primary effect induced by high concentration of αS. Decreasing activity results from synaptic failure rather than neuronal dysfunction. Neuronal death in our study may have occurred secondary to synaptic failure due to insufficient remaining activity. This mechanism suggests that local high concentrations of monomeric αS or perhaps small oligomeric αS species might play an important role in synucleopathies.
